# A comprehensive analysis of phosphatase and tensin homolog deleted on chromosome 10 (*PTEN*) loss in colorectal cancer

**DOI:** 10.1186/s12957-015-0601-y

**Published:** 2015-05-20

**Authors:** Pei-Ching Lin, Jen-Kou Lin, Hung-Hsin Lin, Yuan-Tzu Lan, Chun-Chi Lin, Shung-Haur Yang, Wei-Shone Chen, Wen-Yi Liang, Jeng-Kai Jiang, Shih-Ching Chang

**Affiliations:** Department of Clinical Pathology, Yang-Ming Branch, Taipei City Hospital, Taipei, Taiwan; Division of Colon and Rectal Surgery, Department of Surgery, Taipei Veterans General Hospital, No.201, 2nd section, ShiPai Road, Taipei, Taiwan; Faculty of Medicine, School of Medicine, National Yang-Ming University, Taipei, Taiwan; Department of Pathology, Taipei Veterans General Hospital, Taipei, Taiwan

**Keywords:** Colorectal cancer, Phosphatase and tensin homolog deleted on chromosome 10 (*PTEN*) loss, Mutation, Methylation

## Abstract

**Background:**

Alterations of PTEN, regulator of the PTEN/PI3K-AKT pathway, are common in several types of cancer. This study aimed to do comprehensive analysis of PTEN in colorectal cancer patients.

**Methods:**

Totally, 198 colorectal cancer patients who received surgery at Taipei Veterans General Hospital from 2006 to 2008 were enrolled. Mutations, loss of protein expression, promoter hypermethylation, and DNA copy number of PTEN were analyzed by sequencing, immunohistochemistry, methylation-specific polymerase chain reaction PCR, and quantitative (QPCR), respectively, and correlated with clinicopathological features and patients’ outcome.

**Results:**

Genomic mutations, loss of protein expression, promoter hypermethylation, and decreased DNA copy number of PTEN were found in 4 (2.02 %), 68 (34.3 %), 54 (27.3 %), and 36 (18.2 %) tumors, respectively. Of these 68 tumors with loss expression of PTEN, 34 (50 %) tumors had promoter methylation and 18 (26.5 %) had decreased DNA copy number. All four tumors with PTEN mutations demonstrated loss of PTEN expression. In the stage I disease, frequency of loss of PTEN expression was 20 % and significantly increased to 56.9 % in stage IV disease. Either loss expression of PTEN, PTEN hypermethylation or decreased PTEN copy number was not associated with colorectal cancer (CRC) patients’ outcome.

**Conclusions:**

PTEN alterations were found in up to one-third of colorectal cancers but did not impact CRC patients’ prognosis.

**Electronic supplementary material:**

The online version of this article (doi:10.1186/s12957-015-0601-y) contains supplementary material, which is available to authorized users.

## Background

Colorectal cancer (CRC) is the most common form of cancer and the third leading cause of death in Taiwan [1]. The etiology of CRC from a benign neoplasm, such as a polyp, to a malignant tumor has been well explained by the accumulation of genetic and/or epigenetic alterations, including mutations in oncogenes and tumor suppressor genes [2, 3]. Besides genetic alterations, dysregulation and hyperactivation of several pathways, including the phosphatidylinositol 3-kinase (PI3K) pathway, have been described in several cancers, including CRC [4–6].

PI3K signaling is deregulated through a variety of mechanisms, including the loss of phosphatase and tensin homolog deleted on chromosome 10 (PTEN), located on chromosome 10q23 [7–9]. The frequency of PTEN expression loss is varied and ranges from 4 to 40 % of all CRCs; the loss of PTEN expression has been shown to be associated with disease metastasis [10, 11]. The possible causes of PTEN expression loss include mutation, epigenetic silencing of the PTEN gene through promoter hypermethylation, and a loss of heterozygosity at the PTEN locus [11–15]. However, the relationship between the PTEN alterations and clinicopathologic features in CRC patients was inconclusive because majority of studies focused only one or two aspects of PTEN. Therefore, we undertook a comprehensive molecular analysis of 198 CRC patients with long-term follow-up. We found that 4 (2.02 %), 68 (34.3 %), 54 (27.3 %), and 36 (18.2 %) of the tumors had PTEN mutations, a loss of PTEN expression, promoter hypermethylation, and decreased PTEN copy numbers, respectively. Only the loss of PTEN expression was significantly associated with CRC disease status, but patient’s outcome was not affected by any of PTEN alteration.

## Methods

### Clinical data

Our study enrolled 198 CRC patients who received surgery at Taipei Veterans General Hospital from 2006 to 2008. The exclusion criteria were preoperative radiochemotherapy, emergency operations, and death within 30 days of surgery. The clinical information that was prospectively obtained and stored in the database included the patient’s age, sex, personal and family medical history, location, tumor-node-metastasis (TNM) stage, differentiation, pathological prognostic features [16], and follow-up conditions. Following surgery, patients were monitored quarterly for the first 2 years and semi-annually thereafter. The follow-up protocol included a physical examination, a digital rectal examination, carcinoembryonic antigen (CEA) analysis, a chest radiography, abdominal sonography, and computerized tomography if needed. Positron emission tomography or magnetic resonance imaging was arranged for patients with an elevated CEA level but an uncertain site of tumor recurrence.

### Tumor tissues samples

Before sample collection, written informed consent for tissue collection was obtained from all patients. Tumors were meticulously dissected, and samples were collected from different quadrants of the tumors. Tissue fragments were immediately frozen in liquid nitrogen and stored in the Biobank of Taipei Veterans General Hospital. Sections of cancerous and corresponding normal tissues were reviewed by a senior gastrointestinal pathologist.

### DNA isolation and quantification

After approval by the Institutional Review Board of the Taipei Veterans General Hospital (IRB-2011-11-010IC), frozen tissues and formalin-fixed paraffin-embedded tissue sections (4 μm) were obtained from the Biobank of the Veterans General Hospital for this study. DNA from the tissue specimens was extracted using the QIAamp DNA Tissue Kit (Qiagen, Valencia, CA, USA) according to the manufacturer’s recommendations. The quality and quantity of DNA was confirmed using a Nanodrop 1000 Spectrophotometer (Thermo Scientific).

### Methylation-specific polymerase chain reaction

The PTEN promoter methylation status was examined using the EpiTect Methyl II polymerase chain reaction (PCR) Array [11]. Briefly, input genomic DNA was aliquoted into four equal portions and subjected to mock, methylation-sensitive, methylation-dependent, and double restriction endonuclease digestion. After digestion, the enzymatic reactions were mixed directly with the quantitative PCR (QPCR) master mix and were dispensed into a PCR array plate containing pre-aliquoted primer mixes. The sequences of the primers used for methylation-specific PCR are shown in Additional file 1: Table S1. Real-time PCR was conducted using the specified cycling conditions. Finally, the raw change in the threshold cycle number (∆Ct) was pasted into a data analysis spreadsheet, which automatically calculates the relative quantities of methylated and unmethylated DNA.

### PTEN sequencing

The *PTEN* gene sequencing included the exons and introns adjacent to all of the known splice sites. The primer sequencings are shown in Additional file 1: Table S1. Briefly, the extracted DNA was selectively amplified using PCR and a DNA thermocycler. A no DNA negative control was included in each round of PCR amplification. The PCR products were analyzed using an automated sequencer (ABI Prism 3100 Genetic Analyzer; Perkin-Elmer Applied Biosystems). Each sample was sequenced on both the sense and antisense strands. Each mutation was confirmed by a second round of sequencing on newly synthesized PCR products. By comparing the obtained sequence with the known reference sequence, nonsense, missense, and frameshift mutations were identified.

### *PTEN* DNA copy number

The amount of PTEN was determined using a Perkin-Elmer 7700 TaqMan PCR machine (Perkin-Elmer, Foster City, CA, USA) in duplicate [17, 18]. For *PTEN* copy number analysis, the forward primer sequence was 5′-ACCAGGACCAGAGGAAACCT-3′ and the reverse primer sequence was 5′-GCTAGCCTCTGGATTTGACG-3′. The *PTEN* quantity was corrected using simultaneously measured long interspersed elements (Line-1). The amplification was performed on 10 ng of DNA. The amount of PTEN (2^−ΔCt^) was calculated using the sequence detector software v1.6 (Perkin-Elmer) and is expressed in terms of ΔCt values relative to the quantity of Line-1. The loss of PTEN was defined as a tumor tissue and normal mucosa PTEN ratio less than 50 %. The coefficient of variation (CV) was 3.1 %, and the intra-assay CV was between 0.41 and 1.23 %.

### Immunohistochemical (IHC) analysis

PTEN IHC was performed using the 6H2.1 monoclonal antibody (Cascade Biosciences, Winchester, MA, USA). Briefly, sections were deparaffinized using xylene and progressively rehydrated in graded alcohols. After blocking the endogenous peroxidase activity, sections were incubated with 1 % hydrogen peroxidase in methanol for 30 min, and exposed to a 1:50 dilution of the primary antibody for 1 h. The bound antibody was detected using a biotinylated rabbit anti-mouse immunoglobulin antibody and a horseradish peroxidase conjugated avidin-biotin complex. Immune complexes were visualized with 3,3′-diaminobenzidine. A single section that stained strongly was included in each experiment as a positive control. The negative control consisted of no primary antibody reactions. PTEN expression was evaluated in normal mucosa and adenocarcinomas. PTEN expression was observed in the cytoplasm of CRC tissue. Immunoreactivity for PTEN was interpreted using straightforward clear-cut criteria and was scored as “negative” or “positive”. PTEN protein expression was classified as negative if more than 50 % of the tumor cells showed a loss of expression. In order to assess the PTEN expression in cancer cells, the PTEN expressions in normal mucosa samples, which were included on the same slide, were used as a reference [19].

### Statistical analysis

The statistical endpoint of the analyses was overall survival (OS) from the date of surgery. The OS was calculated from the date of the patient’s operation to the patient’s death. The group distributions for each clinicopathological trait were compared using the two-tailed Fisher’s exact procedure and the *χ*^2^ test. Numerical values were compared using the Student’s *t*-test. Data are expressed as the mean ± the standard deviation. Kaplan-Meier survival curves were plotted and compared using the log-rank test. A multivariate analysis was performed using the Cox proportional hazards model. Statistical significance was defined as *P* < 0.05. Statistical analyses were performed using SPSS software for Windows, version 13.0.

## Results

The patient population was composed of 127 men (64.1 %) and 71 women (35.9 %). The mean age at tumor resection was 67.9 ± 13.2 years (range, 27–80 years; median, 67 years). There were 139 colon cancers and 59 rectal cancers.

### Detection of PTEN alteration

At the genomic level, we analyzed *PTEN* mutation by Sanger sequencing from exon 1 to 9. In total, five mutations were identified in 198 tumors (2.5 %). There were 3 tumors that harbored single point mutations, including G106R, E299Stop and 2 tumors that had a c.265 deletion, which resulted in a frameshift mutation. Protein expression was intact in the tumor with the c.T132C mutation. This mutation was considered to be a single nucleotide polymorphism but was not pathogenic. *PTEN* copy number was analyzed by QPCR, and a low tumor PTEN copy number was defined as a ΔCt difference between the tumor and corresponding normal mucosa, which was greater than 1 cycle. A decreased *PTEN* copy number was found in 36 (18.2 %) of the 198 analyzable primary CRC tumors. Using methylation-specific PCR, we detected two loci in the *PTEN* promoter region, as previously found [11]. There were 54 (27.3 %) tumors that showed *PTEN* promoter hypermethylation. The methylation-specific PCR data was further confirmed to be specific to the *PTEN* gene and not related to a pseudogene, by performing bisulfite sequencing.

**Fig. 1:**
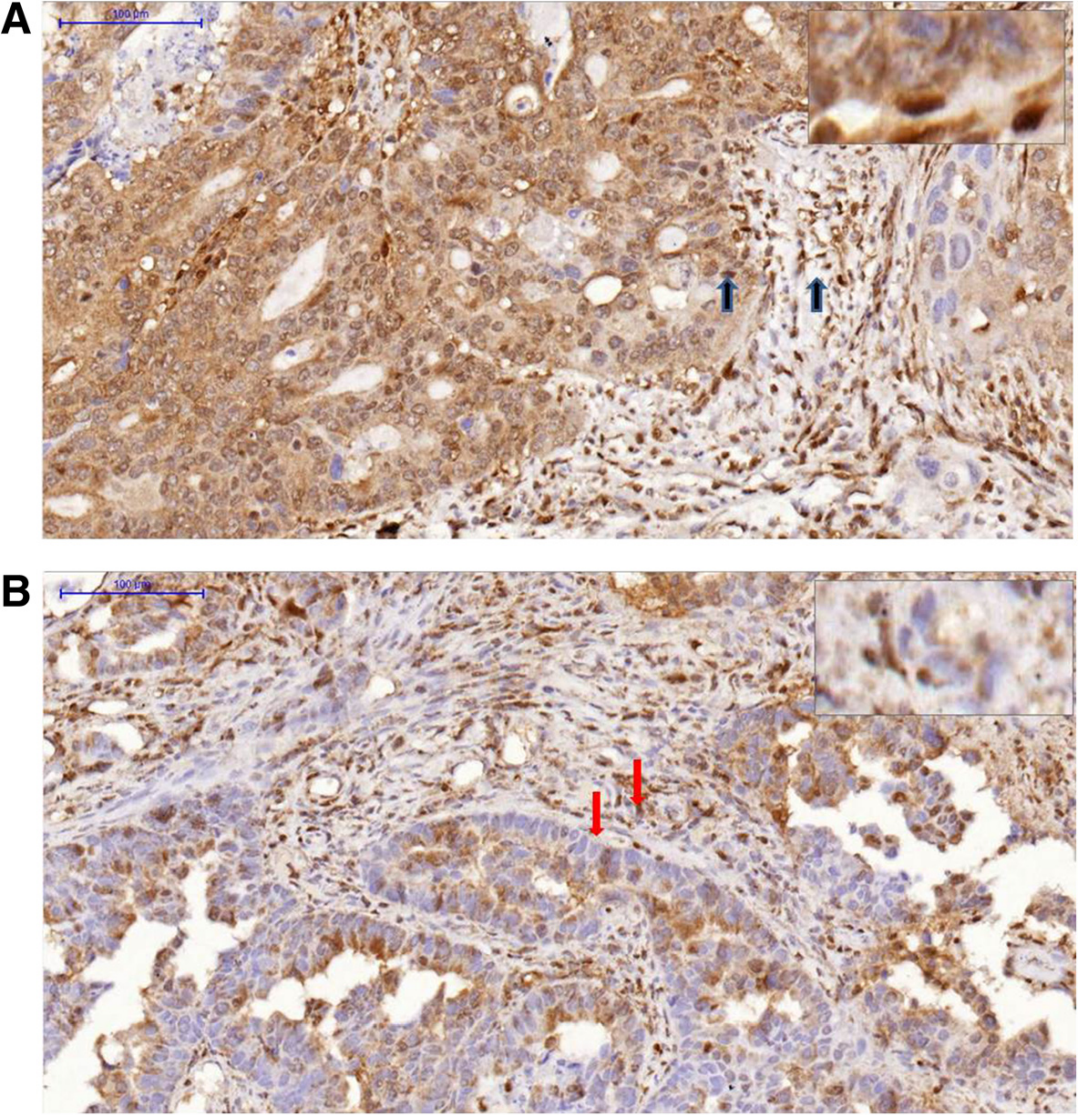
**a** Phosphatase and tensin homolog deleted on chromosome 10 (PTEN) immunohistochemical (IHC) analysis showed protein expression in 80 % of cancer cells. *Arrows* indicate tumor cells with positive staining and reference staining in stromal cells. **b** PTEN IHC analysis showed protein expression in less than 80 % of cancer cells. *Arrows* indicate tumor cells with an absence of staining and positively stained stromal

Of the 198 samples, 68 (34.3 %) demonstrated a loss of PTEN expression (Fig. 1).

Of these 68 tumors with a loss of PTEN expression, 34 (50 %) tumors had *PTEN* promoter methylation and 18 (26.5 %) had decreased *PTEN* DNA copy numbers. The 4 tumors with PTEN mutations, excluding the tumor with the c.T132C mutation, demonstrated a loss of PTEN expression.

### PTEN status and clinicopathological features

As shown in Table 1, the *PTEN* methylation status and change in DNA copy numbers were similarly distributed among patients with different pathological features, including TNM stage, lymphovascular invasion (LVI), and mucinous histology. The loss of PTEN expression was significantly associated with disease stage; in stage I disease, the frequency of PTEN expression loss was 20 % and this significantly increased to 56.9 % in stage IV disease patients. In patients with high CEA levels, 45.6 % of patients had lost PTEN expression, which was significantly higher than the patients with normal CEA levels (28.5 %, *P* = 0.019).

**Table 1:** Clinicopathological features of colorectal cancer with PTEN alteration

		IHC			Methylation			DNA copy number		
		Loss	Intact	*p*	Hyper	Normal	*p*	Decrease	Normal	*p*
	Case no.(%)	68(34.3)	130(65.7)		54(27.3)	144(72.7)		36(18.2)	162(81.8)	
Gender										
Male	127(64.1)	43(33.9)	84(66.1)	0.877	36(28.3)	91(71.7)	0.740	15(11.8)	112(88.2)	0.002
Female	71(35.9)	25(35.2)	46(64.8)		18(25.4)	53(74.6)		21(29.6)	50(70.4)	
Location										
Rectum	61(30.8)	17(27.9)	44(72.1)	0.200	13(21.3)	48(78.7)	0.209	11(18.0)	50(82.0)	0.971
Colon	137(69.2)	51(37.2)	86(62.8)		41(29.9)	96(70.1)		25(18.2)	112(81.8)	
TNM Stage										
I	50(25.3)	10(20.0)	40(80.0)	0.001	18(36.0)	32(64.0)	0.354	11(22.0)	39(78.0)	0.446
II	48(24.2)	13(27.1)	35(72.9)		11(22.9)	37(77.1)		5(10.4)	43(89.6)	
III	49(24.7)	16(32.7)	33(67.3)		14(28.6)	35(71.4)		10(20.4)	39(79.6)	
IV	51(25.8)	29(56.9)	22(43.1)		11(21.6)	40(78.4)		10(19.6)	41(80.4)	
CEA level										
High	68(34.3)	31(45.6)	37(54.4)	0.019	19(27.9)	49(72.1)	0.879	14(20.6)	54(79.4)	0.525
Normal	130(65.7)	37(28.5)	93(71.5)		35(26.9)	95(73.1)		22(16.9)	108(83.1)	
Differentiation										
Poor	14(7.1)	2(14.3)	14(85.7)	0.145	5(35.7)	9(64.3)	0.535	3(21.4)	11(78.6)	0.722
Mod/well	184(92.9)	66(35.9)	118(64.1)		9(26.2)	135(73.4)		33(17.9)	151(82.1)	
LVI										
Present	49(24.7)	21(42.9)	28(57.1)	0.148	14(28.6)	35(71.4)	0.814	11(22.4)	38(77.6)	0.372
Absent	149(75.3)	47(31.5)	102(68.5)		40(26.8)	109(73.2)		25(16.8)	124(83.2)	
Mucinous histology										
Present	20(10.1)	8(40.0)	12(60.0)	0.574	4(20.0)	16(80.0)	0.441	4(20.0)	16(80.0)	0.824
Absent	178(89.9)	60(33.7)	118(66.3)		50(28.1)	128(71.9)		32(18.0)	146(82.0)	

Lymphovascular invasion; numbers in parentheses are percentages

### PTEN status, metastasis, and patient prognosis

All of the patients were followed up for at least 5 years or died of disease before this. In total, 80 (40.4 %) patients had metastatic disease. The metastatic sites included the liver (54), lung (31), peritoneum (12), and bone (6). Of the 80 patients with metastatic disease, 42 (52.5 %) had lost PTEN expression, which was significantly higher than those without metastatic disease (22 %; *P* < 0.001). The frequency of *PTEN* hypermethylation and low *PTEN* copy numbers was similar in patients with and without metastasis. To determine if an alteration in PTEN status had a prognostic value, OS analysis was undertaken. As shown in Table 2, multivariate and univariate analysis, using the Cox regression hazards model, indicated that TNM stage, LVI, CEA levels, and tumor differentiation were all independent prognostic indicators for CRC patients. However, PTEN alterations, including a loss of PTEN expression, *PTEN* hypermethylation, and decreased *PTEN* copy numbers were not associated with CRC patient outcome.

**Table 2:** Univariate and multivariate analysis of overall survival

	Univariate			Multivariate		
Variables	Hazard ratio	95 % CI	*p* value	Hazard ratio	95 % CI	*p* value
Tumor depth (2, 3, 4 vs. 1)	2.43	1.56–4.27	0.004	2.01	1.49–2.78	<0.001
Nodal status (yes vs. no)	3.41	2.25–5.39	0.001	0.001	2.59–4.63	<0.001
Distant metastasis (yes vs. no)	5.56	3.52–9.54	<0.001	4.02	3.72–8.17	<0.001
LVI (yes vs. no)	2.68	1.84–5.39	0.001	2.32	1.35–3.51	0.001
Differentiation (poor vs. well/mod)	2.29	1.32–3.54	0.005	1.78	0.85–2.39	0.307
Preop. CEA (>5 ng/ml vs. <5 ng/ml)	2.87	1.95–4.35	<0.001	2.32	1.45–4.24	0.001
Loss expression of PTEN (yes vs. no)	1.28	0.65–4.52	0.372	1.12	0.87–2.27	0.433
PTEN hypermethylation (yes vs. no)	1.32	0.76–3.51	0.253	1.07	0.73–1.89	0.527
PTEN DNA copy number (high vs. low)	1.11	0.67–2.34	0.652	1.13	0.66–1.78	0.487
Mucinous histology (yes vs. no)	0.96	0.29–3.13	0.949			
Location (rectum vs. colon)	0.75	0.39–1.47	0.406			
Gender (male vs. female)	0.97	0.51–1.86	0.942			

## Discussion

Our study has provided two major contributions regarding the alterations in PTEN status in CRC. Firstly, we found that 2.02, 34.3, 27.3, and 18.2 % of CRCs had genomic mutations, a loss of protein expression, promoter hypermethylation, and decreased DNA copy numbers, respectively. Secondly, a loss of PTEN expression, but not promoter hypermethylation or decreased DNA copy numbers, was shown to be associated with an advanced disease status.

We identified somatic mutations at a low frequency (2.02 %). As the *PTEN* gene has tandem repeat sequences in exons 7 and 8, a higher frequency of *PTEN* mutation was found in microsatellite instability (MSI)-high CRC [10–12, 20, 21]. Our study, in accordance with other unselected series studies, indicated that the frequency of PTEN mutation was relatively low (<5 %) in unselected CRC patients [21–24]. The current CRC somatic mutation frequency in the Catalogue of Somatic Mutations in Cancer database is 5 % [24].

Our presented data demonstrate that a loss of PTEN expression occurs in a significant proportion (34.3 %) of CRCs and is consistent with other reports, which have indicated that 20–40 % of CRCs are negative for PTEN expression [25, 26].

As previous studies have demonstrated [27–29], our series indicated that a loss of PTEN expression was associated with an advanced stage and higher CEA levels. In stage I disease, only 20 % of patients had lost PTEN expression but this significantly increased to 56.9 % in stage IV disease. Furthermore, the intensity of PTEN staining significantly decreased according to the normal–adenoma–carcinoma–metastasis sequence, as shown in a previous study [28]. However, we did not find that a loss of PTEN expression was associated with patient outcome. These conflicting data suggested that a loss of PTEN is a common mechanism of deregulating the PTEN/PI3K-AKT pathway, which plays an important role in the process of CRC tumorigenesis [30, 31] but that sometimes the deregulation of other pathways, such as inactivation of transforming growth factor-β signaling, is required to complete the process [32].

In addition to mutations (2 %), our results indicate that the loss of PTEN protein expression in 68 tumors may have been partly explained by promoter hypermethylation (34; 50 %). Besides our study, previous studies have shown that methylation at the *PTEN* locus may be responsible for a loss of expression in 10–20 % of CRCs, particularly those with an MSI phenotype (11). Furthermore, in accordance with reports that indicate a of loss of heterozygosity plays a role in PTEN loss (12–15), we found decreased *PTEN* gene DNA copy numbers contributed to a loss of PTEN expression in 18 CRC tumors (26.5 %). However, an underlying mechanism could not be identified in almost 40 % of the patients that lost PTEN expression. This discrepancy with previous studies may be due to several factors, including the use of different PTEN antibodies, the IHC scoring, and the subcellular location of PTEN.

## Conclusions

Despite these conflicting results, our study has indicated important PTEN molecular alterations and shown the association between these alterations and the clinicopathological characteristics of CRC. A loss of PTEN expression, originating from genomic alterations, plays some role in the metastatic process of CRC.

## Additional file

Additional file 1: Table S1. Primers-pten. Primers for PTEN sequencing.

## Abbreviations

CEA: carcinoembryonic antigen
CRC: colorectal cancer
CV: coefficient of variation
IHC: immunohistochemistry
LVI: lymphovascular invasion
MSI: microsatellite instability
OS: overall survival
PCR: polymerase chain reaction
PI3K: phosphatidylinositol 3-kinase
PTEN: phosphatase and tensin homolog deleted on chromosome 10
TNM: tumor-node-metastasis
