# Supplementary material for: A comprehensive analysis of phosphatase and tensin homolog deleted on chromosome 10 (PTEN) loss in colorectal cancer
Source: World J Surg Oncol. 2015 May 20;13:186. doi: 10.1186/s12957-015-0601-y (PMC4489205; doi:10.1186/s12957-015-0601-y)
Supplement: Additional file 1: Table S1. — Primers-pten. Primers for PTEN sequencing. [file 12957_2015_601_MOESM1_ESM.docx]

| Primers for PTEN sequencing | |  |
| --- | --- | --- |
|  | PCR/ sequence Primer Name | Primer Sequence (5' to 3') |
|  |  |  |
| 1 | Human PTEN EX1-208F | GCAGCCATGATGGAAGTT |
|  | Human PTEN EX1-1481R | ATCGAAATATGCTCAACCTC |
| 2 | Human PTEN Ex2-30443F | ACATAAATACATTGACCACCT |
|  | Human PTEN Ex2-30897R | GGAGTCCAGGAAATGATATC |
| 3 | Human PTEN EX3-61885F | GAATGACATGATTACTACTCTAAACCCATAG |
|  | Human PTEN EX3-62592R | TCCTAGACAAGACAAGCCACCTAA |
| 4 | Human PTEN Ex4-67355F | AGGCAATGTTTGTTAGTAT |
|  | Human PTEN Ex4-67910R | GTTATGACAGTAAGATACAG |
| 5 | Human PTEN EX5-69433F | CAATACATTATTTTTCTCTGGAATCCAGT |
|  | Human PTEN EX5-70034 | GTGAGGTGATGAATATGTTAAGTAGTTTGA |
| 6 | Human PTEN Ex6-88343F | GTGAAATAACTATAATGGAACAT |
|  | Human PTEN Ex6-88916R | GCTTCTTTAGCCCAATGAG |
| 7 | Human PTEN EX7-94218F | TTCAAACTGGAGAAAATCTTACATTGTT |
|  | Human PTEN EX7-94918R | AATGGAGAAAAAGTGGTTTGTGTCA |
| 8 | Human PTEN EX9-97264F | AACAGATAACTCAGATTGCCTTATAATAGTCT |
|  | Human PTEN EX9-97964R | TCCTACTTCATATCCCAGTCATTTAGC |
| 9 | Human PTEN Ex9-101555F | AGATGAGTCATATTTGTG |
|  | Human PTEN Ex9-102127R | ATGGTGTTTTATCCCTCTTGA |
| Primers for methylation specific PCR | |  |
|  | PTEN-1-129F | TTCGGAGGATTATTCGTCTTCTCCCCATTC |
|  | PTEN-1-377R | AAGAAAAAGGAGGAGAGAGATGGCAGAAGCT |
|  | PTEN-2-102F | GCTGCAGCCATGATGGAAGT |
|  | PTEN-2-302R | AAAAGACGAAGAGGAGGCGAGAA |
